# College Commencements and Alumni Association Meetings

**Published:** 1895-06

**Authors:** 


BUFFALO MEDICAL AND SURGICAL JOURNAL

A MONTHLY REVIEW OF MEDICINE AND SURGERY.

*EDITORS:*

THOMAS LOTHROP, M. D. - - WM. WARREN POTTER, M. D.

All communications, whether of a literary or business nature, should be addressed to the managing editor: 284 FRANKLIN STREET, BUFFALO, N. Y.

VOL. XXXIV. JUNE, 1895. No. 11.

### COLLEGE COMMENCEMENTS AND ALUMNI ASSOCIATION MEETINGS.

### BUFFALO UNIVERSITY MEDICAL COLLEGE.

THE annual commencements of the medical colleges in Buffalo this year fell on dates wider apart than usual. The exercises connected with the forty-ninth annual commencement of Buffalo University Medical College were held Tuesday, April 30, 1895.

The twentieth annual session of the Alumni Association was opened at 10.30 o'clock A. M., in Alumni Hall, at the college building, on High street, under the presidency of Dr. Ernest Wende. After calling the roll, to which a large number of members responded, the annual business meeting began. Among other things, the executive committee was directed to revise the constitution and report its action next year. The chair appointed Drs. Phelps, Jones, Diehl, Meyer and Willard a nominating committee, which subsequently reported the following nominations :

President, Dr. Willis M. Baker, Warren, Pa. ; vice-president, Dr. P. W. Van Peyma, Buffalo ; second vice-president, Dr. D. A. Currie, Englewood, N. J. ; third vice-president, Dr. H. G. Matzinger, Buffalo ; fourth vice-president, Dr. J. A. McPherson, Tonawanda ; fifth vice-president, Dr. J. W. Putnam, Buffalo ; permanent secretary, Dr. E. L. Frost, Buffalo ; recording secretary, Dr. N. Victoria Chappell, Buffalo ; treasurer, Dr. H. U. Williams, Buffalo ; trustees, Dr. H. P. Trull, Williamsville ; Drs. F. E. L. Brecht, E. C. W. O'Brien, Joseph Fowler, M. B. Folwell, Buffalo ; Julius Wenz, Lancaster ; executive committee, Dr. A. A. Jones, Dr. Albert T. Lytle, Dr. F. B. Willard, Dr. Willis Baker ; Dr. John Parmenter, secretary of the faculty.

The afternoon session opened at two o'clock, when Dr. M. D. Mann, dean of the faculty, delivered an address of welcome, which was responded to by Dr. Ernest Wende, the retiring president.

The following scientific papers were then read: Rupture of the Uterus with Prolapse of Intestines after Abortion, M. D. Mann; The Legal Status of Medical Practitioners, Ansley Wilcox; Some Points Concerning the Surgery of the Cranium, Roswell Park; The Treatment of Typhoid Fever by Intestinal Asepsis, A. L. Benedict.

The commencement exercises were held in Music Hall, beginning at eight o'clock in the evening. After prayer had been offered by the Rev. Dr. Andrew Purdy, and a selection played by the orchestra, Dr. John Parmenter, secretary of the faculty, presented the names of fifty-one candidates for graduation, as follows:

Herman J. Abel, Canandaigua; Montressor Axford, Buffalo; Sherman W. Bates, Akron; William J. Boyd, Buffalo; Charles R. Borzilleri, Buffalo; R. V. Crittenden, Ithaca; H. DeGolyer Clapp, Geneva; W. Grant Cooper, Kenyonville; Andrew J. Dick, Watertown; W. J. Deane, Kingston, Ont.; Alex. Geo. Denmark, Shellmouth, Manitoba; A. E. Dean, Brocton; Edward A. French, Rochester; Robert H. Fisher, Spencer; Frederick B. Gould, Buffalo; Frank W. Green, Buffalo; W. G. Grove, Lyons; LeRoy Green, Waterford, Ont.; William House, Buffalo; Geo. W. Hackett, Ceres; O. Grant Harrington, Ripley; Geo. J. Haller, Buffalo; William C. Heussy, Buffalo; John W. Hawes, Buffalo; W. James Howells, Buffalo; Geo. N. Jack, Cameron Mills; Willard Burton Jolls, Dayton; John C. Kamp, Buffalo; Charles C. Kimball, Watertown; C. T. Logan, Warren, Pa.; Henry W. Lattin, Buffalo; S. E. Moore, Waterford, Ont.; Carl C. Mann, Warsaw; B. Ross Nairn, Buffalo; F. R. Pickett, Kenyonville; Frank LeRoy Purdy, Buffalo; Harry A. Powers, St. Johnsbury, Vt.; N. Gorham Russell, Fulton; Edwin C. Reames, Ava; Charles L. Randall, Buffalo; J. H. Robison, Bradford, Pa.; C. B. Rowell, Fairport; Anna M. Stuart, Elmira; Rush B. Shumway, Addison; J. Nelson Shumway, Addison; Chas. H. Sangster, Buffalo; E. J. Smith, Watertown; John Van Doorn, Newark; Sidney D. Wilgus, Buffalo; Christian A. Weinbach, Buffalo; Albert C. Way, Buffalo.

Dr. M. D. Mann, dean, then administered to the class the Hippocratic oath, and the degrees were conferred by the chancellor, Hon. James O. Putnam. The following honors were announced by Dr. Mann:

*Honorable Mention on Standing.*—Sidney D. Wilgus and Robert H. Fisher, equal; H. DeGolyer Clapp, W. J. Deane, W. Grant Cooper, N. Gorham Russell, Evan Jones Smith.

*On Theses.*—George J. Haller, Construction of Modern Hospitals; W. James Howells, The Congestion Treatment of Tubercular Joint Diseases; Sidney D. Wilgus, Differential Diagnosis; William House, Mechanical Treatment of Diarrheal Diseases in Children; Report of a Case of Corea, by H. W. Lattin.

The following graduates were recommended to the trustees for appointment as internes to the General Hospital: Sidney D. Wilgus, W. Grant Cooper, N. Gorham Russell, George J. Haller.

Twenty-seven candidates for the degree of Graduate in Pharmacy were presented by the secretary of the faculty of the pharmaceutical department. The names of twelve persons were also read who had completed the regular examinations, but who lacked the age or length of experience required for graduation. The honors were read, as follows: Edward Francis Kenney, Burton, Ohio; Louis Avery Corning, Buffalo; Charles B. Wheaton, Geneva; Charles Richards, Howard. Junior prize, John G. Brooks, Ithaca; senior prize, Edward Francis Kenney, Burton, Ohio.

Dr. A. P. Southwick, secretary of the faculty of the department of dentistry, read the names of thirty candidates for the degree of Doctor of Dental Surgery. The oath was then administered. The following five graduates carried off the honors in this department: Frederick Jacob Gieser, John James Madden, Lloyd Starr Ingalls, Charles Edwin Flagg, Fred Ellsworth Cloud.

The address to the graduates was then pronounced by the Hon. John G. Milburn, of Buffalo. This eloquent speaker was at his best, seemingly more at home among the doctors than with his own profession. Mr. Milburn's oratory is unique, his rhetoric charming, his periods forcible and telling. To appreciate his address, one should have heard it. He said in part:

I have not come here to weary you with moralizing. It is easy to delineate a profession in such a way as to make failure appear quite certain. But nothing is to be gained by exaggerating the difficulties of life. That there are pitfalls in your pathway is only too true. But there ought also to be that in your nature sufficient to escape from them. High ideals are absolutely necessary to the attainment of the highest ends.

Every man grows along the lines of his fundamental conceptions. If he thinks that life is a failure he will grow morose and cynical. Hope springs from a deep conviction of one's faith in human nature and in human destiny. A man must emerge sometime from the realm of hesitancy and doubt. Strong, clear, affirmative convictions on the rationality of life are needful. There is no occasion for a philosophy of despair or indifference. The extent to which man attains the best that is in him, is the measure of his real happiness. Life is worth the struggle for the best that is in us.

The most perfect life of any organism is secured by the most harmonious development of its varied functions. Human life is no exception to the rule. A man's life should be so ordered as to bring into full play all of his faculties. A man has many sides to his nature. All of these have claims upon him. The intelligent man feels the pressure of these claims and strives to do them justice. The way to bring out the best of which you are capable is to put all of your faculties into active use.

Mr. Milburn closed his address by quoting the maxim, "There is no success worth having that is not based on merit." He urged the students to beware of what passed for success, which was not the real thing. "Finally," said Mr. Milburn, "accept like brave men whatever destiny may have in store for you."

The annual banquet was then held at the Genesee. The tables were artistically decorated, the university colors, blue and white, being conspicuous. Covers were laid for 150. The University Quartette, Messrs. Barrows, Ruffner, Purdy and French were announced to furnish vocal music for the occasion, but owing to the indisposition of one of the number failed to respond to frequent calls by the president and others.

The flow of post-prandial eloquence was in keeping with the general excellence of the arrangements.

Owing to the late hour at which the company began to discuss the viands, following the commencement exercises at Music Hall, it was nearly midnight before the toast-list was fairly reached. The duties of toastmaster were ably discharged by Dr. Ernest Wende, '78, president of the Alumni Association.

Mayor Jewett was called upon to respond to the first toast on the card, "Greater Buffalo," and in so doing outlined the great future that awaits this city with the advent of power from the Falls. His honor made some happy allusions to the past, tracing the wonderful growth of the municipality since the time when the old liberty pole was hoisted on the Terrace, where, happily, it seems destined to remain among our ancient landmarks. But great as has been the city's growth in the past, the evidences are daily accumulating of a still greater future in store for the Queen City of the Lakes.

The Rev. W. B. Gifford responded to "The Two Professions" in an interesting and instructive speech.

Dr. Wm. Warren Potter, responding to "The BUFFALO MEDICAL AND SURGICAL JOURNAL," went back to February 23, 1875, when he delivered the first address to the Alumni Association, in St. James's Hall, which stood on the site now occupied by the Iroquois. The splendid results achieved in the past twenty years proved the wisdom of its founders. Dr. Potter's speech abounded in reminiscences of the old college and the well-remembered men who laid the foundations of what is now a flourishing university, and the story of the JOURNAL's career was exceptionally interesting.

Thomas H. Ramsdell was as witty as usual in his response to "The Physician from a Layman's Standpoint," and Dr. H. A. Powers, '95, did justice to "The Graduating Class."

So ended the most eventful day in the academic year of Buffalo University Medical College.

### NIAGARA UNIVERSITY MEDICAL COLLEGE.

The tenth annual meeting of the Alumni Association of this institution was held on Thursday, May 16, 1895, at the college building on Ellicott street. The president of the association, Dr. E. M. Dooley, of Buffalo, took the chair about 10.30 A. M., and introduced Dr. Herman Mynter, professor of surgery, who delivered an address of welcome, in which he advocated the lengthening of the course of study to four years. The president, Dr. E. M. Dooley, then delivered the annual address, which was a résumé, in part, of the advances in surgical knowledge and methods during the past half century. After the reception of the reports of the treasurer, secretary and executive committee, the election of officers was held with the following result: President, Dr. Joseph J. Kane, A. M., Buffalo; first vice-president, Dr. B. S. Bourne, Hamburg; second vice-president, Dr. D. F. White, Buffalo; secretary, Dr. Henry Osthues, Buffalo; treasurer, Dr. F. M. Boyle, Buffalo; executive committee, Drs. J. J. Mooney, F. A. Hayes and J. J. Finnerty, all of Buffalo.

The afternoon session was called at 3.30 o'clock, when Dr. Bentley S. Bourne, of Hamburg, read a paper entitled Clinical Report of a Country Practitioner. This was followed by a paper by Dr. Herman E. Hayd, of Buffalo, entitled, Intra-peritoneal Operations for Displacements of the Uterus. The special feature of the meeting was the scholarly presentation of the subject, Some

Suggestions in Rectal Surgery, by Dr. Joseph M. Mathews, of Louisville, professor of surgery and clinical diseases of the rectum in the Kentucky School of Medicine. Dr. Mathews is also the author of a treatise on diseases of the rectum, and editor of *Mathews's Medical Quarterly*, a magazine devoted to the consideration of this special branch of surgery. He is a forcible speaker, an able teacher, and his remarks were listened to with the closest attention. The debate on Dr. Mathews's paper was participated in by Dr. Edward Clark, of Buffalo, who practises a like specialty in this city, and who handled the subject in an able manner. We hope to publish Dr. Mathews's paper in full in a future number.

The commencement exercises were held at the Academy of Music and began at 8 o'clock P. M., with a house well filled in every part. Seated upon the stage were the Right Rev. Stephen V. Ryan, chancellor of the University, Dr. John Cronyn, president and Dr. Thomas Lothrop, vice-president of the medical faculty, Dr. Floyd S. Crego and the Rev. P. McHale, president of Niagara University, the speakers of the evening. These were surrounded by other members of the faculty, the board of examiners and invited guests.

The farewell address to the graduates on behalf of the faculty was delivered by Dr. Floyd S. Crego, professor of diseases of the nervous system and insanity. Dr. Crego brought to the attention of the class many problems with which they would be compelled to deal in their professional careers. Especially interesting to the public was his discussion of the question of insanity, its causes and methods of prevention. He complimented the medical department of Niagara University for being among the first institutions to recognize the importance of the study of mental diseases. He gave it as his opinion that, generally speaking, insanity was curable in its first stages. He emphasized the value of early treatment. Dr. Crego also spoke interestingly on the subjects of intemperance and crime, treating them from the pathological standpoint.

After the administration of the college oath by Dr. John Cronyn, the degrees were conferred by the chancellor, Bishop Ryan, according to the usual custom adopted in this University—namely, hooding. This interesting ceremony may be briefly described as follows:

The candidates are presented to the chancellor in a Latin introduction, which recites the fact that the candidate is entitled to the degree of Doctor of Medicine, and then each kneels before the chancellor, who holds the candidate's hands in his and recites the Latin phrase conferring the degree, while an alumnus of the college invests him with the crimson hood, which was worn by the graduates present upon the stage.

The following are the members of the graduating class:

John Anderson, Toronto, Ont.; William James Austin, Niagara Falls; Bernard Hugh Brady, North Java; Marshall Clinton, Buffalo; Arthur Victor Conley, Buffalo; Edward James Cook, Buffalo; John Havemeyer Daniels, Buffalo; James Sandwick Dawson, Little Current, Ont.; Peter Adolphus Edelsward, Buffalo; Anna Earl Hutchinson, North Evans; Richard Kimpton, Buffalo; William Costigan Lewin, Buffalo; Murray F. Mudge, Gasport; George Marion Nye, Wellsville; Charles Augustus O'Dea, Erie, Pa.; William Priess, Tuscarora, Ont.; Bion Elmer Smith, Angola; Henry Smoyer, Pendleton Center.

The three gentlemen having the highest standing in the final examinations were Mr. Daniels, first; Mr. Conley, second; Mr. Lewin, third.

Each candidate was applauded when his name was called and when the chancellor conferred the degree. To Miss Anna Earl Hutchinson, of North Evans, the woman member of the class of '95, was given a perfect ovation when her name was read. The applause continued as she walked upon the stage and did not cease until she stood before Chancellor Ryan to receive her degree.

When the degree had been conferred, the Very Rev. P. McHale, president of Niagara University, arose to address the graduating class. His address was able, timely and scholarly. He criticised the modern trend of thought as empirical and as resting upon no solid foundations. He argued that there could be no permanent conflict between science and religion, since the sources of both were the same. The religious factor could never be eliminated from the factors that made for human progress. Christianity must enter into every rational scheme of education. It was written indelibly across the true civilization of the day. Mr. McHale spoke eloquently on the advantages of a liberal education, and closed by welcoming the graduates to the ranks of those who devote themselves to the highest interests of civilization.

Bishop Ryan spoke a few words of congratulation to the members of the class upon the successful completion of their course.

At the close of the ceremonies at the Academy of Music, the faculty, alumni and invited guests repaired to the Hotel Iroquois, where the annual banquet was held. The tables, prepared for about 100 people, were well filled by 10 o'clock, when a well-appointed dinner was served in the main dining-room of the hotel.

Occupying a prominent seat was Dr. Anna E. Hutchinson, of the class of '95, the first woman graduate of the medical department of the University. Other seats on the right of the president of the alumni, who acted as toastmaster, were occupied by Drs. Cronyn, Lothrop and Banta. On his left sat Dr. Joseph M. Mathews, of Louisville, Hon. E. B. Jewett, mayor, Dr. W. W. Potter and Dr. Wm. H. Pitt. After doing ample justice to the dinner and while coffee and cigars were serving, Dr. Dooley, the toastmaster, arose and in appropriate remarks introduced Mayor Jewett, who responded to the toast, The City of Buffalo. The Mayor called attention to the fact that on the basis of exports and imports Buffalo stands the sixth commercial city of the world, being outstripped only by London, Liverpool, New York, Hamburg and Chicago. He spoke of Buffalo as the "Electric City," and said that with the increasing importance of the manufacturing industries, the population of the city might be confidently expected to be well on toward the million mark by the end of the present century.

The Rev. Father Grace responded to Old Niagara, and spoke entertainingly on the Cataract and the College. George H. Kennedy, of the faculty of the law department, spoke on Law, especially considered from the viewpoint of medical jurisprudence. The Hon. T. V. Welch, superintendent of the state reservation at Niagara Falls, spoke on Nature, M. D. His address was humorous and interesting. Old Kentucky inspired Dr. Joseph M. Mathews, of Louisville, to an impassioned flight of oratory, which was generously applauded, and Dr. Henry Smoyer, of '95, spoke to the toast of The Class of '95.

It was long after midnight when the guests separated and the early hours of Friday, May 17, 1895, witnessed the closing scenes of one of the most enjoyable commencement days in the history of Niagara University Medical College.

---

CHRISTIAN Science and Faith Cure are having a struggle for existence in New Hampshire, as a bill is now pending before the legislature making their practice illegal and punishable by fine.—*Chicago Medical Recorder.*

